# Biotic threats for 23 major non-native tree species in Europe

**DOI:** 10.1038/s41597-021-00961-4

**Published:** 2021-08-06

**Authors:** Elisabeth Pötzelsberger, Martin M. Gossner, Ludwig Beenken, Anna Gazda, Michal Petr, Tiina Ylioja, Nicola La Porta, Dimitrios N. Avtzis, Elodie Bay, Maarten De Groot, Rein Drenkhan, Mihai-Leonard Duduman, Rasmus Enderle, Margarita Georgieva, Ari M. Hietala, Björn Hoppe, Hervé Jactel, Kristjan Jarni, Srđan Keren, Zsolt Keseru, Marcin Koprowski, Andrej Kormuťák, María Josefa Lombardero, Aljona Lukjanova, Vitas Marozas, Edurad Mauri, Maria Cristina Monteverdi, Per Holm Nygaard, Nikica Ogris, Nicolai Olenici, Christophe Orazio, Bernhard Perny, Glória Pinto, Michael Power, Radoslaw Puchalka, Hans Peter Ravn, Ignacio Sevillano, Sophie Stroheker, Paul Taylor, Panagiotis Tsopelas, Josef Urban, Kaljo Voolma, Marjana Westergren, Johanna Witzell, Olga Zborovska, Milica Zlatkovic

**Affiliations:** 1 Institute of Silviculture, University of Natural Resources and Life Sciences, Vienna (BOKU), Peter-Jordan Str. 82, 1190 Wien, Austria; 2 European Forest Institute, Platz der Vereinten Nationen 7, 53113 Bonn, Germany; 3 Forest Entomology, Swiss Federal Research Institute WSL, Zürcherstrasse 111, 8903 Birmensdorf, Switzerland; 4 ETH Zurich, Department of Environmental Systems Science, Institute of Terrestrial Ecosystems, 8092 Zurich, Switzerland; 5 Forest Protection, Swiss Federal Research Institute WSL, Zürcherstrasse 111, 8903 Birmensdorf, Switzerland; 6 Faculty of Forestry, University of Agriculture, Al. 29 Listopada 46, 31-425 Kraków, Poland; 7 Forest Research, Forestry Commission, Northern Research Station, Roslin, EH25 9SY Great Britain; 8 Natural Resources Institute Finland, Luke, Latokartanonkaari 9, 00790 Helsinki, Finland; 9 FEM Research and Innovation Centre, Fondazione Edmund Mach, Via E. Mach 1, 38010 San Michele all’Adige, Italy; 10 The EFI Project Centre on Mountain Forests MOUNTFOR, Via E. Mach 1, 38010 San Michele all’Adige, Italy; 11 Forest Research Institute, Hellenic Agricultural Organization Demeter, Vassilika, 57006 Greece; 12 Walloon Public service (SPW), 23 av Maréchal Juin, 5030 Gembloux, Belgium; 13 Slovenian Forestry Institute, Vecna pot 2, 1000 Ljubljana, Slovenia; 14 Institute of Forestry and Rural Engineering, Estonian University of Life Sciences, Fr. R. Kreutzwaldi 5, 51006 Tartu, Estonia; 15 Faculty of Forestry, “Ștefan cel Mare” University of Suceava, Universității Street 13, 720229 Suceava, Romania; 16 Institute for Plant Protection in Horticulture and Forests, Julius Kuehn Institute (Federal Research Centre for Cultivated Plants), Messeweg 11/12, 38104 Braunschweig, Germany; 17 Department of Entomology, Phytopathologyy and Game fauna, Forest Research Institute - Bulgarian Academy of Sciences, St. Kliment Ohridski 132, 1756 Sofia, Bulgaria; 18 Department of Fungal Plant Pathology in Forestry, Agriculture and Horticulture, Norwegian Institute of Bioeconomy Research (NIBIO), Innocamp Steinkjer, skolegata 22, 7713 Steinkjer, Norway; 19 Institute for National and International Plant Health, Julius Kuehn Institute (Federal Research Centre for Cultivated Plants), Messeweg 11/12, 38104 Braunschweig, Germany; 20 Biodiversité, Gènes et Communautés (BioGeCo), French National Institute for Agriculture, Food, and Environment (INRAE), University Bordeaux, F-33610 Cestas, France; 21 Department of Forestry and Renewable Forest Resources, Biotechnical Faculty, University of Ljubljana, Vecna pot 83, 1000 Ljubljana, Slovenia; 22 Faculty of Forestry, University of Banja Luka, Bulevar vojvode Stepe Stepanovica 75A, 51000 Banja Luka, Bosnia and Herzegovina; 23 Forest Research Institute, National Agricultural Research and Innovation Centre, Farkassziget 3, H-4150 Püspökladány, Hungary; 24 Department of Ecology and Biogeography, Nicolaus Copernicus University, Lwowska 1, PL-87-100 Toruń, Poland; 25 Centre for Climate Change Research, Nicolaus Copernicus University, Lwowska 1, PL-87-100 Toruń, Poland; 26 Institute of Plant Genetics and Biotechnology SAS, Akademicka 2, P. O. Box 39A, SK-950 07 Nitra, Slovakia; 27 Unidade de Xestión Ambiental e Forestal Sostible, Universidade de Santiago de Compostela, Campus de Lugo, 27002 Lugo, Spain; 28 Laboratory of Environmental Toxicology, National Institute of Chemical Physics and Biophysics (NICPB), Akadeemia tee 23, 12618 Tallinn, Estonia; 29 Faculty of Forest Science and Ecology, Agriculture Academy, Vytautas Magnus University, Studentu 11, Akademija, 53361 Kaunas, Lithuania; 30 Mediterranean Facility, European Forest Institute, Sant Pau Art Nouveau Site, Sant Antoni M. Claret 167, 08025 Barcelona, Spain; 31 Centro di Ricerca Foreste e Legno, Council for agricultural research and analysis of the agricultural economy (CREA), Viale Santa Margherita, 80, 52100 Arezzo, Italy; 32 Norwegian Institute of Bioeconomy Research (NIBIO), P.O. Box 115, NO-1431 Ås, Norway; 33 “Marin Drăcea” National Research-Development Institute in Forestry, Station Câmpulung Moldovenesc, Calea Bucovinei, 73bis, 725100 Câmpulung Moldovenesc, Romania; 34 EFI Atlantic, European Forest Institute, 69, Route de Arcachon, F-33610 Cestas, France; 35 IEFC Institut Européen de la Forêt Cultivée, 69, Route de Arcachon, F-33610 Cestas, France; 36 Department of Forest Protection, Austrian Federal Research Centre for Forests, Natural Hazards and Landscape (BFW), Seckendorff-Gudent-Weg 8, 1131 Vienna, Austria; 37 Centre for Environmental and Marine Studies (CESAM) & Department of Biology, University of Aveiro, 3810-193 Aveiro, Portugal; 38 Coillte Unit 27, Coillte Forest, Danville Business Park, Kilkenny, R95 YT95 Ireland; 39 Department of Geosciences and Natural Resource Management, University of Copenhagen, Rolighedsvej 23, DK-1958 Frederiksberg C., Germany; 40 UCD Forestry, School of Agriculture and Food Science, University College Dublin, UCD Forestry, School of Agriculture and Food Science, University College Dublin, D04 V1W8 Dublin, Ireland; 41 Forest Research, Forestry Commission, Northern Research Station, Roslin, Midlothian, EH25 9SY Great Britain; 42 Institute of Mediterranean Forest Ecosystems, Hellenic Agricultural Organization “Demeter”-, Terma Alkmanos, 11528 Athens, Greece; 43 Faculty of Forestry and Wood Technology, Mendel University, Zemědělská 3, 613 00 Brno, Czech Republic; 44 Siberian Federal University, Svobodnyy Ave, 79, 660041 Krasnoyarsk, Russia; 45 Institute of Forestry and Rural Engineering, EstonianUniversity of Life Sciences, Kreutzwaldi 5, 51006 Tartu, Estonia; 46 Southern Swedish Forest Research Center, PO Box 49, SE-230 53 Alnarp, Sweden; 47 Polissya Branch, Ukrainian Research Institute of Forestry and Forest Melioration, Neskorenych st. 2, Dovzhik, Ukraine; 48 Institute of Lowland Forestry and Environment (ILFE), University of Novi Sad, Antona Cehova 13d, 21 000 Novi Sad, Serbia

**Keywords:** Forestry, Forest ecology

## Abstract

For non-native tree species with an origin outside of Europe a detailed compilation of enemy species including the severity of their attack is lacking up to now. We collected information on native and non-native species attacking non-native trees, i.e. type, extent and time of first observation of damage for 23 important non-native trees in 27 European countries. Our database includes about 2300 synthesised attack records (synthesised per biotic threat, tree and country) from over 800 species. Insects (49%) and fungi (45%) are the main observed biotic threats, but also arachnids, bacteria including phytoplasmas, mammals, nematodes, plants and viruses have been recorded. This information will be valuable to identify patterns and drivers of attacks, and trees with a lower current health risk to be considered for planting. In addition, our database will provide a baseline to which future impacts on non-native tree species could be compared with and thus will allow to analyse temporal trends of impacts.

## Abstract (summary)

**Table:** 

Measurement(s)	area of attack of enemy species on non-native tree • intensity of attack of enemy species on non-native tree
Technology Type(s)	species identification • visual observation method
Factor Type(s)	plant health • country • species
Sample Characteristic - Organism	tree
Sample Characteristic - Environment	forested area
Sample Characteristic - Location	Europe

Machine-accessible metadata file describing the reported data: 10.6084/m9.figshare.14345921

## Background & Summary

Hundreds of tree species have been introduced to Europe over the last few hundred years. Initially, non-native tree species were mainly introduced out of curiosity and for ornamental purposes but a considerable number of non-native tree species have also been tested and used for forestry and restoration purposes^1^. Today, at least 150 non-native trees are grown in European forests with varying importance and area occupied in different regions and countries^2^. The higher productivity of some non-native trees compared to native tree species or the tolerance of poor soil conditions have been dominating factors in the choice of non-native trees over the last two hundred years. More recently, the search for alternative tree species that can survive and perform well under climate change conditions has become a topic of high importance in forestry^1^. However, also the concern about potential spread and negative environmental impact of alien species is increasing^1^. In order to share the extremely diverse knowledge and experience about the benefits, disadvantages or difficulties of growing non-native trees in Europe and to bring together proponents and opponents of non-native trees, the COST Action FP1403 (NNEXT) – ‘Non-native tree species for European forests: experiences, risks and opportunities’ was initiated. This European project ran from 2014–2018 and involved 36 member countries.

**Fig. 1:**
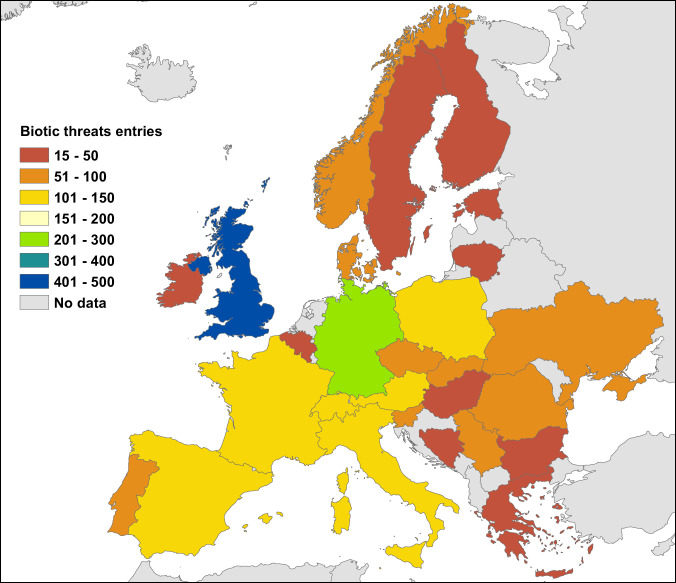
Number of entries in the biotic threats database by country.

Due to their non-European origin and thus lacking common evolutionary history, particular non-native tree species traits related to plant defence e.g. secondary metabolites in their leaves, may cause different responses of native and introduced pests and pathogens compared to native trees. Moreover, due to their limited distribution, the forest health situation and dynamics are different to those of native tree species. According to the enemy release hypothesis^3^ non-native trees may be more vital and productive because their natural enemies are absent and thus give them a competitive advantage. However, a lack of adaptation of non-native trees to European pests and pathogens could pose a high risk of attack for non-native trees. In the course of the NNEXT project, we compiled country specific information on observed biotic threats for non-native trees and their impacts for 27 countries (Fig. 1). The data sources utilised by the entomologists and pathologists of each country encompassed international scientific and grey literature, national forest health databases and national and regional reports. Besides, we also allowed for expert knowledge/observations to be integrated in the assessment of the biotic threats. The information compiled in this database has a high practical and scientific value. Practitioners intending to test new non-native trees may wish to learn about European or invasive insect pests, pathogens or other organisms, which have been observed to impact a specific non-native tree in other European countries and the extent and type of damage encountered. On the scientific side, a lot remains to be learned about the dynamics of pest and pathogen impacts on non-native trees. Previous studies have shown that at the continental scale the main drivers of insect and pathogen attacks on non-native trees are the abundance of a non-native tree, the presence of congeneric species and the time since introduction^4,5^. The limitations of those studies are the low spatial resolution (continental scale) or small study extent and the limited consideration of the extent of damage caused by insects and pathogens. With the data presented here it will be possible to investigate whether drivers of and mechanisms underlying the variability in the level of damage might differ at a finer spatial resolution. By providing important baseline information the data will also serve to compare future data with and thus allow to analyse temporal trends of impacts of pest species on non-native trees. Not least this database allows to identify trees with a lower current health risk to be considered for planting, while we acknowledge that the plant health situation is not static and new or more severe attacks may occur for different reasons, e.g., through imported or naturally arriving non-native pests or pathogens, or caused by climate change (e.g. through better breeding conditions of pests) or by additional host shifts, which may occur with considerably prolonged cultivation time or area of cultivation^4,5^.

## Methods

We designed a Microsoft Excel spreadsheet that allowed for straightforward recording of the occurrence of the biotic threats and their overall impact on specific non-native trees in a country. The required information was NNT (non-native tree), COUNTRY (for which the information was provided), ORGANISM_GROUP, ORDER, FAMILY, GENUS, scientific NAME of the biotic threat, AUTHOR of the taxon name, ORIGIN (continent of origin of the biotic threat), main host species (latter omitted), 1ST_OBSERVATION (year of 1^st^ observation of the damage), PRIM_DAMAGE and SEC_DAMAGE (primary damage, which is the symptom most detrimental to the tree health, and, if any, secondary type of damage, which is an additional symptom), LEVEL of impact on an individual tree, MAX_AREA (maximum continuous area impacted), AGE_CLASS (tree cohort where impact occurs), CONFIDENCE level for the provided information, REF (references), COMMENTS (latter omitted) and DATA_PROVIDER (name and email address). We predefined selection options for the following fields: ORGANISM_GROUP, ORIGIN (multiple selections allowed), PRIM_DAMAGE and SEC_DAMAGE (primary and secondary type of damage), LEVEL, MAX_AREA and AGE_CLASS (Table 1). Because we received multiple entries for PRIM_DAMAGE and mainly for SEC_DAMAGE we transformed these columns in the final table into single columns for all eight damage types where ‘0’ means not observed, ‘1’ observed and ‘2’ observed and originally filled as primary type of damage. To describe the overall data quality, the data providers could choose among three CONFIDENCE levels on the type and severity of impact/damage (‘high’ - Reliable/high quality data sources on impact. The case was reviewed and verified by an expert; ‘medium’ - Reliable/medium quality data sources of impact. The impact was either reported by a reliable forester but could not be reviewed and verified by an expert or it was published in a professional journal, but it is not clear from the publication that it was checked by an expert; ‘low’ - Low quality data sources of impact. Observation was reported by a non-professional or reported in a non-professional journal, without being confirmed by an expert; Table 2). Any missing information was indicated with NA. In the final table, one line/entry was allowed per biotic threat per non-native tree per country. This procedure was chosen as the middle ground between requiring a detailed description of every single attack incidence (not feasible) and simple occurrence recording of a biotic threat on a non-native tree (limited value).

**Table 1:** Selection options (bold) and supplementary explanations (italic) for the columns PRIM_DAMAGE, SEC_DAMAGE, LEVEL, MAX_AREA, AGE_CLASS.

PRIM_DAMAGE/SEC_DAMAGE: *The primary/secondary functional or structural type of damage to the tree*
**Flowers**: *Damage to the inflorescence or cones*
**Seeds**: *Damage to the immature or ripe seeds, either on the plant or when already on the ground*
**Buds/shoots**: *Damage to the buds of shoots or leaves or to non-lignified branches*
**Foliage**: *Damage to the foliage which impairs its function*
**Bark/cambium/phloem**: *Damage to the outer layer of the tree from the vascular cambium outwards*
**Wood/xylem**: *Structural damage to the wood/xylem, e.g., wholes, weakening, breakage*
**Roots**: *Damage to the roots*
**Seedlings/saplings**: *Damage to the whole seedling/sapling*
**LEVEL**: ***Level of impact for a healthy tree***
**1** - **no effect**: Noticeable impact/damage but no effect on individual tree fitness
**2** - **reversible effect**: Effects on individual tree fitness which are reversible (within 5 years)
**3** - **irreversible effect**: Changes to individual tree fitness which are irreversible (within 5 years)
**4** - **death**: Individual tree dies
**MAX_AREA**: ***Maximum (continuous) area affected***
**1** - **individual trees**: *Only individual trees are affected, neighbouring trees are healthy*
**2** - **group of trees**: *A group of trees (<0.5* *ha) is affected, but the rest of the stand is unaffected*
**3** - **forest block**: *A forest block (0.5* *ha - 100* *ha) is affected, but the rest of the area is unaffected*
**4** - **large forest block**: *A large forest block (100* *ha - 10,000* *ha) is affected, but the rest of the area is unaffected*
**5** - **landscape**: *A very large continuous forest area (>10,000* *ha) is affected*
**6** - **all trees**: *+/− all trees in the country affected, irrespective if single trees or continuous areas*
**AGE_CLASS**: ***Development stage when impact typically occurs***
**1** - **juvenile trees**: *Young trees unable to produce seeds*
**2** - **adult trees**: *Mature trees potentially able to produce seeds*
**3** - **all**: *Young and mature trees*

Please mind that the columns PRIM_DAMAGE and SEC_DAMAGE were dropped in the final table and replaced with single columns for all eight damage types where 0 means not observed, 1 observed and 2 observed and originally filled as primary type of damage.

**Table 2:** Confidence level categories and how often they have been chosen.

Confidence levels	*Number of cases*
High - *Reliable/high quality data sources on impact. The case was reviewed and verified by an expert*.	1786
Medium - *Reliable/medium quality data sources of impact. The impact was either reported by a reliable forester but could not be reviewed and verified by an expert or it was published in a professional journal, but it is not clear from the publication that it was checked by an expert*.	189
Low - *Low quality data sources of impact. Observation was reported by a non-professional or reported in a non-professional journal, without being confirmed by an expert*.	265
NA	64

The tree species to be investigated in this study were selected mainly based on their importance in silviculture and the area they occupy^2^, but also some less widely distributed species were included. In total, we requested biotic threats information for 24 non-native trees. One subjected non-native tree, *Acacia melanoxylon* with only one entry from Spain, was discarded due to limited data.

We approached forest damage experts in all 36 European member countries of NNEXT. Country representatives in NNEXT were either themselves forest protection experts or they contacted experts at universities or national forest research stations/institutes. Twenty-nine countries responded to our request to fill the biotic threats database, but only 27 countries eventually filled the database in the requested manner.

The information on the year of 1^st^ observation turned out to be difficult to verify and thus we received many NAs or ambiguous information. Several improvement steps were taken. If  “<” than a specified year had been entered, we changed it to the specified year (e.g. <1850 was changed to 1850). If a period was indicated, we changed it to the mean year (e.g. 1890’s was changed to 1895). If any text had been entered that could not be interpreted in a way to yield a certain year, we changed this to NA (e.g. ‘several times’ or ‘since introduction of a non-native tree’). To fill NAs, we took a look at reference of the entry, and whenever the reference was a publication specific to this biotic threat in that country, we took the year of the publication as a substitute. To distinguish such data from the original information on the year of 1^st^ observation received from the data providers, we added the column YEAR_ADDED indicating with 1/0 whether the year was filled from the references or not.

The columns on the type of damage appeared to be filled very heterogeneously by our national experts and left many gaps. We thus decided to homogenize this information across countries based on current knowledge on species autecology and considering the information provided by the country experts. The damage information was also restructured. Instead of primary and secondary type of damage, we introduced eight columns for the eight possible types of damages, where the observation of a type of damage was given as 0 (not observed), 1 (observed) or 2 (observed and filled as primary type of damage by data providers, see above). This was done by the three database managers (one forest entomologist, one forest pathologist and one general forest ecologist). Although one particular pest or pathogen species could cause damage to different parts of the same tree in different countries (e.g. an insect that attacks different tree parts during larval and adult stage), we decided to use a common classification of all eight types of damage across countries because of the above-mentioned inconsistent data provided by country experts, but still allowed for a weighting of the primary damage in each country. While this approach will not allow for testing fine scale differences in damage types caused on a particular tree species across Europe, it will still allow for coarse differences in main impacted tissue among countries and identifying traits of species that might increase the probability of attack. Furthermore, the LEVEL and MAX_AREA of damage provided by our database still varies among countries, allowing for tests on the main drivers of damage variability across Europe, e.g. depending on county-specific differences in the number of congeneric tree species, time since introduction of the non-native tree and area of the non-native tree, which have been shown to be main drivers at the European scale^4,5^.

The database managers added two additional columns. A column SPECIALISATION was introduced to differentiate the host plant niche breadth of species based on the main higher plant lineages^6^ following the approach published by Gossner *et al*.^7^. An organism was assigned ‘monophagous’ if it attacks species of one genus, 'oligophagous' if it attacks species of one higher plant lineage (i.e., bryophytes, ferns, gymnosperms, angiosperms: monocots, angiosperms: basal eudicots, angiosperms: eurosids, angiosperms: euasterids) and 'polyphagous' if it attacks species from more than one higher plant lineage. The column RELATIVE_ORIGIN categorises the biotic threats into species native to Europe but not at the origin of the non-native tree (‘Europe’), species from same origin as the tree species and not native in Europe (‘origin’), species native in both, Europe and the home range of the non-native tree (‘both’) and species from another region, neither Europe, nor the origin of the non-native tree (‘third’).

## Data Records

We provide a database on synthesised biotic threat information for 23 non-native tree species (*Abies grandis, Abies nordmanniana, Acer negundo, Ailanthus altissima, Cedrus atlantica, Chamaecyparis lawsoniana, Cryptomeria japonica, Eucalyptus camaldulensis, Eucalyptus globulus, Fraxinus pennsylvanica, Juglans nigra, Larix kaempferi, Larix sibirica, Picea sitchensis, Pinus contorta, Pinus radiata, Pinus strobus, Populus x canadensis, Prunus serotina, Pseudotsuga menziesii, Quercus rubra, Robinia pseudoacacia, Thuja plicata*) in 27 European countries. The table contains 2304 entries. The database is available at Figshare^8^ in a comma separated values (.csv) format. The data table consists of 28 columns: ID, NNT (non-native tree species), COUNTRY (for which the data are provided), ORGANISM_GROUP, ORDER, FAMILY, GENUS, NAME (scientific name), AUTHOR (author of the scientific name), ORIGIN (continent of origin of the biotic threat), 1ST_OBSERVATION (year of 1^st^ observation), YEAR_ADDED (1 indicates where the year in 1ST_OBSERVATION was filled based on the reference year of publication), eight columns for the different types of damage (BUDS_SHOOTS, FOLIAGE, BARK_CAMBIUM_PHLOEM, WOOD_XYLEM, ROOT, SEEDLINGS_SAPLINGS), LEVEL (level of impact on an individual tree), MAX_AREA (maximum continuous area impacted), AGE_CLASS (tree cohort where impact occurs), CONFIDENCE (confidence level for the provided impact information), SPECIALISATION, RELATIVE_ORIGIN, REF (references), DATA_PROVIDER (name and email address). The full list of references associated with column REF of the data table are provided in a second .csv file^8^.

## Technical Validation

All country tables were checked by the database managers for formal correctness of the information provided. For example, entries for fungi occurring only on dead plant material or in association with ectomycorrhiza were removed, because they are not the topic of this data collection. The database managers checked all scientific names, families and orders and changed them where necessary to the current accepted name. Fungal nomenclature is particularly complex due to sexual (teleomorph) and asexual (anamorph) states having different names. The new codex rules adopted at the International Botanical Congress in 2011 advocated the abandoning of dual naming system for pleomorphic fungi (“one fungus, one name” convention)^9^. Therefore, we took the currently valid name from the online databases for fungal nomenclature, Indexfungorum.org and MycoBank.org, matched with the most recent relevant taxonomic literature. For animals we used the Fauna Europaea^10^ as baseline and adapted recent changes. ORIGIN was completed and corrected by the database managers.

Two rounds of data quality checks involving the data providers were included in the data acquisition procedure. We calculated simple descriptive statistics for every country to determine the number of entries per non-native tree, the number cases where for a tree occurring in a country (based on NNEXT information^2,11^) we did not have any biotic threats entries in the database and the number of entries per organism group and non-native tree (compare Table 3). In online-only Table 1 we provide the number of entries per tree species per country. The pre-analysis helped to indicate missing or biased information. A short, country specific report on these findings (stating i.e. whether there was a considerable and unexpected imbalance between insect pests and pathogens and for which non-native trees occurring in the country no records had been provided) was sent to the data providers. The report was accompanied with a formal request to (i) provide a personal assessment of the quality and completeness of the data (bias assessment), (ii) to name options to improve the data quality, (iii) to add new entries and (iv) to fill missing information in existing database entries. For the assessment of a potential bias in the number of entries and level of detail provided for different tree species for example due to missing experts or lacking economic value of a tree species resulting limited recording and knowledge on attacks, we offered the following categories: 1 – ‘The data well reflect the situation of the pest/pathogen impact. There is no bias due to prioritization of certain tree species and/or lack of experts’; 2 – ‘The data on pest/pathogen impact have some bias. The bias due to prioritization of certain tree species and/or lack of experts is, however, minor’; 3 – ‘The data on pest/pathogen impact have major bias. Due to prioritization of certain tree species and/or lack of experts the data does not reflect the complete situation in the country and thus should not be used in a cross-country analysis’; This call for data quality check and completion was successful and led to a large number of new and completed entries. In a second round of quality check, the data providers were given a final chance to update the database and give a final assessment of the data quality/bias (Table 4).

**Table 3:** NNT - Number of non-native tree species out of the 23 investigated tree species known to occur in a country; NA - Number of cases where non-native trees are known to occur in a country, but are without biotic threats entry in our database; Number of database entries for pathogens, insects and other organisms groups per country and total number of entries per country.

Country	NNT	NAs	Pathogens	Insects	Other	Total number of entries
AT	13	1	51	50	12	113
BA	4	1	6	9	0	15
BE-WAL	4	7	13	11	0	24
BG	9	4	14	16	0	30
CH	11	1	43	78	6	127
CZ	14	1	52	38	4	94
DE	18	0	94	125	25	244
DK	12	3	22	33	0	55
EE	8	0	17	10	4	31
ES	10	2	39	63	3	105
FI	6	1	11	28	4	43
FR	15	7	28	59	15	102
GB	18	1	290	120	4	414
GR	8	1	14	24	1	39
HU	1	11	18	13	7	38
IE	7	2	14	6	2	22
IT	14	5	57	42	8	107
LT	8	1	12	7	2	21
NO	10	0	47	17	11	75
PL	10	7	25	105	2	132
PT	8	0	33	18	1	52
RO	8	6	17	45	4	66
RS	15	0	43	45	9	97
SE	8	1	10	21	2	33
SI	14	1	37	44	1	82
SK	15	3	28	53	2	83
UA	8	4	9	49	2	60

**Table 4:** Data quality assessment by the data providers concerning a potential bias in insect pests and pathogens, and steps taken thereafter (contacting of new experts and completing the database).

Country	Initial data quality assessment			After initial assessment			Final assessment
	Bias in insects and other pests	Bias in pathogens	Potential for more data from new experts	Contacting of new experts	Response of new experts	New entries/updates/gap filling	Overall bias
**AT**	2	2	No	n.a.	n.a.	Yes	1
**BA**	1	1	No	n.a.	n.a.	No	1
**BE**-**WAL**	2	2	Yes	Yes	No	Yes	2
**BG**	1	1	No	n.a.	n.a.	Yes	1
**CH**	1	1	No	n.a.	n.a.	Yes	1
**CZ**	1	1	Yes	No	n.a.	Yes	1
**DE**	NA	NA	Yes	Yes	Yes	Yes	2
**DK**	3	3	Yes	Yes	Yes	Yes	1
**EE**	2	2	Yes	Yes	Yes	Yes	1
**ES**	1	1	No	n.a.	n.a.	Yes	1
**FI**	NA	NA	No	n.a.	n.a.	Yes	1
**FR**	2	2	Yes	Yes	Yes	Yes	2
**GB**	2	2	Yes	Yes	No	No	1
**GR**	1	1	No	n.a.	n.a.	Yes	1
**HU**	1	1	No	n.a.	n.a.	No	1
**IE**	1	1	Yes	No	n.a.	Yes	1
**IT**	NA	NA	No	n.a.	n.a.	Yes	1
**LT**	2	2	No	n.a.	n.a.	Yes	2
**NO**	1	1	No	n.a.	n.a.	Yes	1
**PL**	NA	NA	No	n.a.	n.a.	Yes	1
**PT**	2	2	Yes	Yes	No	Yes	2
**RO**	2	3	Yes	Yes	Yes	Yes	1
**RS**	1	1	No	n.a.	n.a.	Yes	1
**SE**	3	3	Yes	Yes	Yes	Yes	1
**SI**	1	1	No	n.a.	n.a.	Yes	1
**SK**	NA	NA	Yes	No	n.a.	No	2
**UA**	2	2	Yes	Yes	Yes	Yes	2

Every data provider agreed to check for new records and fill NAs in existing entries. The bias is evaluated according to the following scheme: 1 – ‘The data well reflect the situation of the pest/pathogen impact. There is no bias due to prioritization of certain tree species and/or lack of experts’; 2 – ‘The data on pest/pathogen impact have some bias. The bias due to prioritization of certain tree species and/or lack of experts is, however, minor’; 3 – ‘The data on pest/pathogen impact have major bias. Due to prioritization of certain tree species and/or lack of experts the data does not reflect the complete situation in the country and thus should not be used in a cross-country analysis’;

n.a. – not applicable.

## Online-only Table

**Online-Only Table 1:** Number of entries per non-native tree species and country. NA indicates tree species growing in a country, but without biotic threats entry in the database.

Country	Abies grandis	Abies nordmanniana	Acer negundo	Ailanthus altissima	Cedrus atlantica	Chamaecyparis lawsoniana	Cryptomeria japonica	Eucalyptus camaldulensis	Eucalyptus globulus	Fraxinus pennsylvanica	Juglans nigra	Larix kaempferi	*Larix sibirica*	Picea sitchensis	Pinus contorta	Pinus radiata	Pinus strobus	Populus x canadensis	Prunus serotina	Pseudotsuga menziesii	Quercus rubra	Robinia pseudoacacia	Thuja plicata
**AT**	13	20	2	2		10				3	6						6	13	NA	16	8	3	11
**BA**										NA				1			5			6		3	
**BE**-**WAL**	7					NA						7		NA			NA	1	NA	9	NA	NA	NA
**BG**	NA		1	NA	NA	2				1	1					4	5	NA		5	5	6	
**CH**	6	14		5	8	6						9					10	NA		29	14	11	15
**CZ**	16	2	2	NA		1	2				1				1		18	2	1	34	3	6	5
**DE**	11	5	4	3	6	7	1			2		12		16	4		31	7	5	47	17	57	9
**DK**	5	15			1	NA				1		4		9	6		2	NA	NA	7	3	1	1
**EE**										1		6	12	1	2		2			6		1	
**ES**			NA	NA	3	3		5	29		1					28		17		10	4	5	
**FI**		1										3	8		27		1	NA		3			
**FR**	5	2	2	NA	5	NA	NA	1	3		NA	4	2	13	NA	NA	1	15	2	22	23	2	NA
**GB**	18	12	4	3	14	33	8				2	34		88	38	35	11	NA	1	37	30	20	26
**GR**			1	1	1			3	NA							4		24		2		3	
**HU**		NA	NA	NA		NA				NA	NA						NA	NA	NA	NA	NA	38	
**IE**	NA					1						5		8	3	1	1			3			NA
**IT**		NA	6	8	1	1	1	27	7		5	NA		NA		4	1	15	1	13	NA	18	NA
**LT**			5										4		1		3	1	NA	3	2	2	
**NO**	3	22				2						5	5	11	18		1			6			2
**PL**	13	9	NA	NA		NA				NA	NA	16	10	10	25		26		NA	19	2	NA	2
**PT**						4	1	7	29							3				2	1		5
**RO**		NA	2	NA		NA				1	NA	NA					4	34	NA	11	3	10	1
**RS**	1	1	1	1	5	5				5	2			5			7	34		10	5	6	9
**SE**	1											2	1	3	21		1			3	NA		1
**SI**	1		NA	4	3	4					2	2		1	3		19	20		9	6	7	1
**SK**	NA	8	7	NA		2	3			1	NA		1	4	1		6	5	1	10	9	20	5
**UA**		4	2		6						NA	3					10	5	NA	NA	NA	28	2
